# Targeted alpha therapy for glioblastoma

**DOI:** 10.3389/fmed.2022.1085245

**Published:** 2022-12-16

**Authors:** Jolanta Kunikowska, Alfred Morgenstern, Kacper Pełka, Frank Bruchertseifer, Leszek Królicki

**Affiliations:** ^1^Department of Nuclear Medicine, Medical University of Warsaw, Warsaw, Poland; ^2^European Commission, Joint Research Centre (JRC), Karlsruhe, Germany; ^3^Laboratory of Center for Preclinical Research, Department of Methodology, Medical University of Warsaw, Warsaw, Poland

**Keywords:** glioblastoma, glioma, alpha therapy, substance P, actinium, bismuth, SP, GB

## Abstract

According to the 2021 World Health Organization Classification of Tumors of the Central Nervous System, glioblastoma (GB) is a primary brain tumor and presents with the worst prognosis. Due to its infiltrating characteristic, molecular heterogeneity, and only partly preserved function of the blood-brain barrier, the median overall survival time is short (9–15 months), regardless of comprehensive treatment including surgery, radiotherapy, and chemotherapy. Several novel treatment strategies are under investigation. Unfortunately, none of them produced successful results; 90% of patients have a recurrence of the disease within 6 months. Local administration of the drug could be a promising approach to delivering treatment with minimized side effects, due to the recurrence of 95% glioblastomas in a margin of 2 cm at the primary site. Several ligand-receptor systems have been evaluated, such as targeting tenascin, the extracellular matrix protein, or radiolabeled somatostatin analogs, as it is overexpressed with the SSTR-2 receptor system in around 80% of gliomas. Moreover, this study revealed that the NK-1 receptor is overexpressed in GB, suggesting that substance P (SP) may serve as a ligand. A variety of radioisotopes, beta- (^131^I, ^90^Y, or ^177^ Lu) and alpha emitters (^213^Bi, ^225^Ac, or ^211^At), with different physical properties were tested for treatment. Alpha particles have many advantages over beta radiation such as short range with higher linear energy transfer. According to that characteristic, it is extremely dose delivered to the targeted cells, while reducing harm to nearby healthy tissue. Additionally, the biological effect of alpha radiation is independent of the cell cycle phase, cell oxygenation and O-6-methylguanine-DNA methyltransferase (*MGMT*) gene promoter methylation status. In this article, we summarize the experience with local treatment of primary and secondary GBs with locally used radioisotopes such as [^213^Bi]Bi-DOTA-SP or [^225^Ac]Ac-DOTA-SP.

## Introduction

The most aggressive primary brain tumor with the worst prognosis is glioblastoma multiforme [called glioblastoma (GB) since 2016], according to the World Health Organization (WHO) Classification of Tumors of the Central Nervous System (CNS). At the end of Louis et al. (1) WHO in the fifth edition of CNS introduced significant changes to the tumor entities and classification.

Currently, the diagnosis of GB requires the recognition of genetic changes such as no mutation of isocitrate dehydrogenase 1 and 2 (IDH-wild type) and no mutation in histone 3 (H3-wildtype), which makes it impossible to classify the entries as not otherwise specified (NOS). In this article, based on the classification used in the discussed studies, we use the nomenclature from 2016.

Glioblastoma originates from the glial cells, which are the supportive tissue in the brain. GB grows expeditiously, and therefore, it infiltrates the surrounding healthy brain tissue. Consequently, the entire tumor is nearly impossible to excise. Moreover, a single tumor consists of different types of cells. Hence, a drug targeted at specific cells may not work on the other cells.

The survival rate in patients with GB is low, i.e., approximately 40% in the first-year post-diagnosis and 17% in the following year (2, 3). The standard treatment includes surgery, radiotherapy (RT), and chemotherapy with a median overall survival time of only up to 9–15 months (2, 3).

Therefore, it is necessary to search for new drugs and different forms of treatment. Distributing medicament to the destined area is also challenging. The blood vessels in the CNS are impenetrable to toxins and diseases from the blood. This blood-brain barrier protects the brain and spinal cord. Nonetheless, it also prevents many drugs from penetrating into GB. In that scenario, the possibility of finding local administration seems attractive.

To fulfill the concept of theragnostics, target and applied isotopes should be defined.

## Target

Several targeting vectors have been used during glioma treatment.

Their mechanism of action is a criterion of their classification:

– Targeted molecular therapies: *BRAFV600* mutation, EGFR, Exportin-1, EGFR mutations, mTOR, VEGF; the angiogenesis and its mediators such as VEGF seem to be particularly attractive targets.
– DNA-damaging agents, including RT and cytotoxic chemotherapy. A significant direction in the improvement of these methods increases their influence on cancer cells, saving the healthy ones. One of the innovative approaches is also targeting the tumor-specific DNA repair mechanism.

Targeting tumor metabolism. The data show that the regulators of GB metabolism can be used as prognostic, diagnostic, and perhaps also therapeutic tools aimed at facilitating the choice of glioblastoma treatment. Observations from several studies indicate that tumor genotype and the brain’s biochemical and cellular microenvironment shape the metabolic reprogramming of glioblastoma cells, which also informs the decisions about the choice of targeted treatment.

– Immunotherapies (EGFR peptide vaccinate, anti-dendritic cell vaccinate, viral therapies, and nivolumab–checkpoint inhibitor).

Transforming growth factor-β (TGF-β) is a key molecule, which is responsible for glioblastoma-mediated immunosuppression; programmed cell death protein 1 (PD-1) is neutralizing antibodies to immune checkpoint molecules and is now leading in the field of cancer immunotherapy.

In the previous studies, some of the targets were clinically evaluated for targeting radionuclide therapy of glioma (4–8).

Zalutsky et al. (7) first evaluated the antibodies targeting tenascin as a biologically active peptide that significantly contributes to angiogenesis in glioblastoma. Tenascin activity in stem cell niches and the central nervous system was highlighted. A relationship has been demonstrated between the grade of malignancy and the expression of tenascin C. Moreover, tenascin may be one of the factors influencing the plasticity of cancer cells, i.e., the mutual conversion of transformed non-stem cells into cancer stem cells. In addition, tenascin may play the role in cancer cell plasticity, i.e., in the reciprocal conversion of transformed non-stem cells to cancer stem cells.

Thereafter, Merlo et al. and Schumacher et al. (4, 8) used the overexpression of the SSTR-2 receptor system as an approach for targeted treatment. In total, 80% of glioma tumors have been shown to express SSTR-2, which is particularly often in grades II and III and less often in grade IV (4). Human macrophages are characterized by especially the expression and upregulation of SSTR2, including tumor-associated macrophages (TAMs) and/or microglia. These components account for up to 40% of GB cells. TAMs foster the growth of malignant glioma by secreting proangiogenic factors, generation of a local immunosuppressive microenvironment, and stimulating invasion through the production of interleukin (IL)-10, TGF-β, matrix metallopeptidase-9, and vascular endothelial growth factor.

Substance P (SP), the main ligand of neurokinin type 1 receptor (NK-1) which is consistently overexpressed in all gliomas irrespective of the degree of malignancy, was first applied by Kneifel et al. (5). NK-1 receptors were also identified on the cells of tumor-infiltrating the intertumoral and peritumoral vasculature (9). SP is an undecapeptide composed of the amino acid chain Arg-Pro-Lys-Pro-Gln-Gln-Phe-Phe-Gly-Leu-Met, with an amidation at the C-terminus. The vector has a low molecular weight of only 1.8 kDa, which is a sufficient condition and distribution between tumors after local injection. This characteristic allows for rapid diffusion in the brain and renders radiolabeled SP analogs, which makes them very promising candidates for the local treatment of glial tumors.

## Isotopes

The energy, range of radiation, and type of emission are critical in targeted radionuclide therapy. These parameters play a crucial role in the radiobiological process in which the final effect is the death of tumor cells. We could divide the radioisotopes into two types, namely, β-emitting isotopes: ^131^I, ^90^Y, and ^177^Lu and α-emitting isotopes: ^225^Ac, ^213^Bi, and ^211^At.

In the first pilot study for glioma treatment, the β^–^ emitters including ^90^Y and ^177^Lu were used (the detailed physical parameters are listed in Table 1) (5). The range of both is millimeters (for ^177^Lu 3–4 mm, and for ^90^Y up to 10 mm), thus taking into the consideration, “cross-fire effect” and the brain as a critical organ–with tumors located near very sensitive areas and responsible for physiological process structure–the range appears to be too long. Another disadvantage of β^–^ emitters is that even in high-energy emitting conditions, they are not powerful enough to induce the double-strand breaks of DNA, but rather work by the indirect effects.

**TABLE 1:** The detailed physical parameters of isotope use for local glioblastoma treatment.

	^177^Lu	^90^Y	^213^Bi	^225^Ac
Half-life	6.71 day	64.0 h	46 min	9.9 day
Radiation	β−, γ (17%)	β−	α, β−	α
Mean beta energy	133 keV	933 keV	435 keV	
Range in water	0.25 mm	4.3 mm	1.45 mm	
Maximum beta energy	497 keV	2,284 keV	1,422 KeV	
Range in water	1.9 mm	11.8 mm	6.65 mm	
Maximum alpha energy			8.4 MeV*	5.9 MeV
Range in water			85 μm*	47 μm

*Energy and range of alpha particle emitted by ^213^Po daughter nuclide.

In contrast to that scenario, alpha-emitting radionuclides have distinguishing features that may be useful in the targeted therapy, like a relatively small range of impact (<100 μm) and high linear energy transfer (LET ≈100 keV/μm). In human tissue, those advantages led to providing therapeutic doses to targeted cells while limiting the harm to the surrounding non-cancerous tissue. Alpha radiation has a predominantly direct effect on cell death because it induces double-strand breaks of DNA, occurring along the trajectory of densely ionizing particles, and it is largely independent of the cell oxygenation status and the cell cycle phase (10–13). Of the several alpha emitters suitable for use in anti-cancer therapy, the pair of radionuclides derived from ^225^Ac to ^213^Bi generators have proven to be particularly promising. The preferred chemical attributes of the trivalent metalions Ac(III) and Bi(III) allow obtaining a solid connection to biomolecules using the common DOTA chelate molecules (1,4,7,10-tetraazacyclododecane-1,4,7,10-tetraacetic acid).

## Clinical studies with targeted radioisotope therapy for glioma

The main indications for local targeted treatment in glial tumors are as follows:

• Critically located primary brain tumor.
• Recurrent primary brain tumor.
• Recurrent secondary brain tumor.

The first pilot study to provide the proof of principle that there is a suitable and specific distribution of the radiopharmaceutical to the tumor was performed on a group of 20 glioma patients with WHO grades II–IV. The patients had a local intratumoral injection of radiolabeled SP (5). Initially, most of the patients (18/20) were treated with the SP labeled with beta emitters ^90^Y and ^177^Lu. Only in a subset of two patients with critically located tumors, the alpha emitter ^213^Bi was used to reduce the “crossfire effect.” Stable disease or improvement in neurological conditions was observed in the majority of the patients (13/20). The toxicity of treatment was limited only to one patient with symptomatic radiogenic edema.

Another study focused on the usage of the intertumoral injections of [^90^Y]Y-DOTAGA- SP as a neoadjuvant treatment before the surgery in patients with GB. The majority of patients (15/17) had stabilization or improvement in their functional status. Neoadjuvant therapy of glioblastoma with locally injected [^90^Y]Y-DOTAGA-SP is feasible and has low toxicity. Moreover, it was helpful to achieve a prognostically significant degree of resections (14).

To increase the delivery of energy and minimize the “crossfire effect,” future works should be focused on the alpha emitters.

The first pilot study of local injections of [^213^Bi]Bi-DOTA-[Thi8,Met(O2)11]-substance P ([^213^Bi]Bi-DOTA-SP) was performed in patients with critically localized gliomas (15). Treatment was well tolerated by all patients, and the follow-up MRI suggested radiation-induced necrosis and tumor demarcation. Some of the patients underwent subsequent resection, which confirmed necrosis in histopathological validation. Although the injection was done to critically locate gliomas, no neurologic deficit was observed. The study found that targeted local radiotherapy with [^213^Bi]Bi-DOTA-SP may be an ingenious and beneficial treatment strategy for malignant gliomas that are unfavorably located, as primarily no surgical gliomas can be resected after the intertumoral radioisotope treatment.

The study for recurrent glioma tumor grades II-IV with [^213^Bi]Bi-DOTA-SP has been carried out at the Medical University of Warsaw.

The analysis of 18 patients with the recurrence of primary glioma grade IV treated with [^213^Bi]Bi-DOTA-SP after standard treatment demonstrated favorable survival parameters: the PFS was 3.7 months and OS was 8.5 months, measured from the start of radioisotope treatment. The median overall survival from the start of the primary diagnosis (OS-d) was 21.5 months and the median survival from the diagnosis of the recurrence (OS-r) was 9 months (16).

Secondary glioblastoma has different genetic characteristics compared to primary tumors and evolves out of the low grade or anaplastic astrocytoma precursor lesions. This has implications for the differences in clinical presentation and survival times. In secondary glioblastoma (transformation from grade II/III to grade IV), [^213^Bi]Bi-DOTA-SP treatment showed that median PFS, OS-t, and OS-d was 13.6, 16.4, and 46.8 months, consecutively (17). Better results were obtained in patients with several [^213^Bi]Bi-DOTA-SP doses of injections, which may be due to cumulatively a larger dose of treatment and/or finer clinical condition at the start of the treatment.

The limited supply and high cost of large ^225^Ac/^213^Bi generators required for targeted alpha therapy with ^213^Bi-SP led to the investigations using SP labeled with the longer-lived mother nuclide ^225^Ac requiring significantly lower activities.

The first dose escalation [^225^Ac]Ac-DOTA-SP treatment study with three subgroups 10, 20, and 30 MBq administered activity showed that therapy was well tolerated with only mild and transient side effects (epileptic seizures, edema, and aphasia) up to 30 MBq per cycle (18). Thrombocytopenia grade 3 was observed only in one patient treated with 30 MBq. There were no other grade 3 and 4 toxicities related to [^225^Ac]Ac-DOTA-treatment in all groups. However, surprisingly, the calculated survival parameters were comparable to [^213^Bi]Bi-DOTA-SP with OS-d 35.0 and OS-r/c 13.2 months. From the beginning of treatment with [^225^Ac]Ac-DOTA-SP, the median PFS was 2.4 months, while OS-t was 9.0 months. No statistically significant differences have been found between the investigated dose escalation groups.

The assessment of factors influencing therapy with [^213^Bi]Bi-DOTA-SP and [^225^Ac]Ac-DOTA-SP is still ongoing.

The ^212^Pb–another alpha emitter–is currently being used in phase 1, a non-randomized, open-label, dose-escalation study of ^212^Pb-octreotate in adult subjects with neuroendocrine tumors (NET) overexpressing somatostatin receptors (19). Preliminary results presented during EANM, SNMMI, and ASCO congresses in 2021 and 2022 showed a high percentage of overall response rate (ORR), i.e., 83.3% per RECIST 1 (in five out of six subjects).

To our knowledge, currently, there are no other ongoing or published studies on the use of ^212^Pb- in the management of patients with glioma.

## Future perspective

As the local radioisotope procedure should still be considered experimental, the place of this procedure in the treatment regimen needs to be analyzed. The following options can be evaluated:

(a) The therapy can be used as a rescue treatment option in relapsing patients.
(b) Potentially, therapy can be started right after the relapse is diagnosed.
(c) The therapy might be used as an adjuvant therapy right away after finishing the primary treatment.
(d) The therapy might be used as a neoadjuvant therapy before the surgery.

**FIGURE 1:**
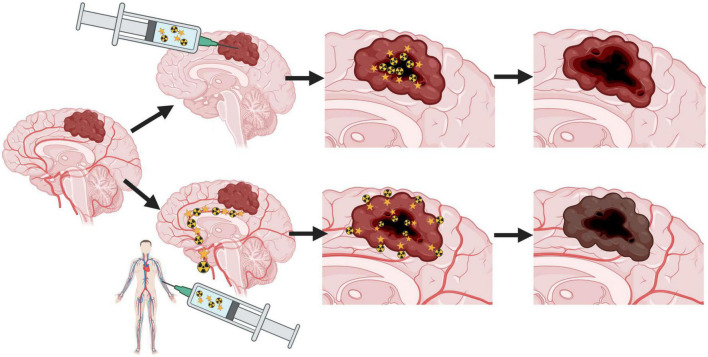
The idea of theranostic treatment of glioblastoma with local and intravenous injection of radiopharmaceutical.

Due to the negative features of glial tumors such as diffuse and very rapid progression, new strategies should be applied. Even local injection can give satisfactory results with prolongation of survival parameters in comparison to standard treatment. Probably, the most promising will be the finding of the right target, which will allow intravenous delivery of radioisotopes to every, even peripheral, part of the tumor (Figure 1).

## Author contributions

JK, LK, and KP conceived and wrote the manuscript. AM and FB critically revised the manuscript for intellectual content. All authors edited and commented on the original manuscript, and read and confirmed the final manuscript.
